# Finding the ‘QR’ to Patient Safety: Applying Gamification to Incorporate Patient Safety Priorities Through a Simulated ‘Escape Room’ Experience

**DOI:** 10.7759/cureus.4014

**Published:** 2019-02-05

**Authors:** Xiao Chi Zhang, Gretchen Diemer, Hyunjoo Lee, Rebecca Jaffe, Dimitrios Papanagnou

**Affiliations:** 1 Emergency Medicine, Thomas Jefferson University, Philadelphia, USA; 2 Internal Medicine, Thomas Jefferson University, Philadelphia, USA; 3 Emergency Medicine, Stony Brook University Hospital, Stony Brook, USA

**Keywords:** medication error reporting, medical simulation, graduate medical education, gamification, escape room, advedrse event reporting, innovation

## Abstract

Medical errors are the eighth leading cause of mortality in the United States and contribute to over one million preventable injuries. In an effort to prevent medical errors, reporting systems serve as invaluable tools to detect patient safety events and quality problems longitudinally. Historically, trainees (i.e., students and residents) rarely submit incident reports for encountered patient safety threats. The authors propose an immersive learning experience utilizing gamification theory and leveraging the increasingly popular ‘escape room’ to help resident trainees identify reportable patient safety priorities.

All 130 incoming intern physicians at the Thomas Jefferson University (Jefferson) were enrolled in the Patient Safety Escape Room study as part of their residency orientation (June 2018). The residents were randomly divided into 16 teams. Each team was immersed in a simulated escape room, tasked with identifying a predetermined set of serious patient safety hazards, and successfully manually entering them into the Jefferson Event Reporting System within the time allotted to successfully ‘win the game’ by ‘escaping the room’. Quick response (QR) codes were planted throughout the activity to provide in-game instructions; clues to solve the puzzle; and key information about patient safety priorities at Jefferson. All participants underwent a formal debriefing using the feedback capture grid method and completed a voluntary post-study survey, adapted from Brookfield’s Critical Incident Questionnaire (CIQ). The study was IRB exempt.

Thematic analysis of the post-activity CIQ survey (*n *= 102) revealed that interns were engaged during the immersive learning experience (*n *= 42) and were specifically engaged by having to independently identify patient safety threats (*n *= 30). Participants identified team role assignment (*n *= 52) and effective communication (*n *= 26) as the two most helpful actions needed to successfully complete the activity. Participants were overall surprised by the success of the education innovation (*n *= 45) and reported that it changed how they viewed patient safety threats. Areas for improvement include clearer game instructions and using a more streamlined event reporting process.

The escape room patient-safety activity allowed interns to actively engage in an innovative orientation activity that highlighted the importance of patient safety hazards, as well as providing them with the opportunity to document event reports in real-time. Next steps will include longitudinally tracking the quantity of error reports entered by this cohort to determine the effectiveness of this educational intervention.

## Introduction

In the United States, medical errors are the eighth leading cause of mortality, with an estimated 44,000 to 98,000 unnecessary deaths occurring each year, and over one million preventable injuries [1]. Estimated medical errors have resulted in nearly $30 billion of lost income secondary to disability and additional healthcare costs [2]. Specific medical error risk factors include inexperienced clinicians (i.e., postgraduate trainees), introduction to new procedures, prolonged hospital stays, complex patient care, and extremes in age [3]. Despite numerous national and institutional efforts to prevent medical errors, reporting errors through mandatory or voluntary means (i.e., patient safety event reporting systems) remain an invaluable tool in identifying and tracking patient safety events and threats to clinical quality. In the groundbreaking report released by the Institute of Medicine’s (IOM) report, ‘To Err is Human: Building a Safer Health System’, the authors found that more than 90% of errors were preventable, and the successful reporting of these errors could provide critical data that would inform future efforts to improve patient safety [4-6].

Mandatory error reporting systems for serious patient injuries and/or death aim to 1) ensure further investigation and follow-up; 2) provide incentives to avoid potential penalties and public exposure; and 3) ultimately improve patient safety [4]. Voluntary reporting systems, in contrast, are based on a passive form of surveillance, requiring personnel directly involved in a near-miss event to provide essential information and details [4]. The latter system has the benefit of capturing true events or near misses that would assist in investigating and learning more about the circumstances surrounding the event [4].

Despite the confidentiality that comes with voluntary reporting, there are numerous limitations and barriers to effective, voluntary event reporting. Common physician barriers for submitting incident reports include: 1) lack of incident follow-up; 2) long forms; 3) perceived ‘trivial’ nature of the incident; 4) lack of time; and 5) unclear responsibility for making the report [7]. Furthermore, increased public pressure on preventable errors, coupled with the threat of legal action and additional psychological sequelae (i.e. guilt, anger, inadequacy, and depression) associated with these errors, can perpetuate a culture of blame and avoidance, and deter helpful voluntary incidental reporting [8-10]. While both reporting systems are integral for detection and improvement of existing infrastructure weaknesses, efforts are required to encourage the use of voluntary reporting system.

Clinician educators are tasked with teaching physicians-in-training medical and patient safety practices. Teaching patient safety and error reporting to the Graduate Medical Education (GME) audiences, however, is challenging. Only a minority of event reports are contributed by resident physicians and fellows at Thomas Jefferson University (Jefferson). In the setting of the aforementioned barriers to reporting, the authors also recognize that a new, unfamiliar system would potentially further deter new interns from reporting events. 

In effort to augment active learning and engagement with teaching voluntary event reporting, the authors developed an interactive, immersive learning experience incorporating gamification and leveraging the increasingly popular ‘escape room’ design to incoming Jefferson intern and resident physicians to better identify potential patient safety priorities and practice logging these events into the Jefferson Event Reporting System. ‘escape rooms’ have risen in popularity in creating a high-fidelity, foreign environment that rewards players for working together, solving clues, and completing successions of mind-bending tasks in order to ‘escape the room’ in a limited amount of time. Notably, the immersive and adaptive nature of the escape room has enabled this team-building activity to be incorporated as an innovative educational tool for a multitude of medical specialties [10-15].

We propose that exposing incoming interns and residents to various team-based activities on critical patient safety priorities, as well as providing them with the opportunity for guided reflection during a facilitated debriefing, will allow them to 1) identify local patient safety priorities; 2) physically enter an event report using the Jefferson Event Reporting System; and 3) practice using teamwork skills to address patient safety threats.

## Technical report

Study setting

Four standard simulation rooms at the Rector Clinical Skills and Simulation Center at Thomas Jefferson University in Philadelphia, Pennsylvania (PA) were secured to create four separate escape rooms that were operated concurrently: two of these rooms simulated a hospital inpatient room, and the other two rooms simulated a patient room in the emergency department (ED). The activity took place in June 2018, during new intern and resident orientation.

Study participant selection

All participants were interns (i.e., post-graduate year one, PGY-1, residents) beginning their training at Thomas Jefferson University Hospitals. There were no exclusion criteria. All participants consented to participate in the activity. All incoming PGY-1 residents were asked to attend this activity as part of their orientation curriculum prior to starting their respective residency programs.

Materials required

Participants were not asked to bring any additional tools or materials to the escape room. Specific equipment required for the escape rooms has been listed in Table 1.

**Table 1:** Equipment and materials required for the patient safety escape room simulation cases

Patient Safety Escape Room Materials
Hospital bed
Mannequin
Computer (with internet connection to enter error report)
Ventilator
Sequential compression devices
Isolation cart with gowns
Opened lumbar puncture kit (with sharps removed for safety)
Identification bracelet
Falls risk bracelet
Allergy bands
Adult diaper
Yellow falls socks
Urinal
Restraints
Incentive spirometer
Quick response (QR) codes
Door sign
Paper chart
Clipboard with blank list for recording hazards and clues (Appendix: hazard list)

Personnel requirements

In order to coordinate, facilitate, and set-up the rooms for each session, 12 faculty members were recruited to supervise the events. There were four simulation facilitators, four simulation observers, two traffic controllers, and two debriefing facilitators. Simulation facilitators were present in each of the rooms to provide any necessary hints, suggestions, or technical support to effectively guide the learners through the cases. Observers were able to remotely view all of the student activity in a control room via video streaming through an encrypted, institution wireless network. Traffic controllers helped organize and coordinate learners moving from room to room. The debriefing facilitators were tasked with debriefing teamwork skills used during the activity, as well as discussing patient safety hazards noted in the rooms.

Group structure

All 130 incoming interns were randomly divided into 16 teams, each comprising seven to eight residents. Teams were tasked to work together to identify a predetermined series of patient safety threats that were planted in each simulated escape room, and successfully enter these events into the Jefferson Event Reporting System within the allotted time of 20 minutes in order to successfully ‘escape the room’ and win the game.

**Figure 1:**
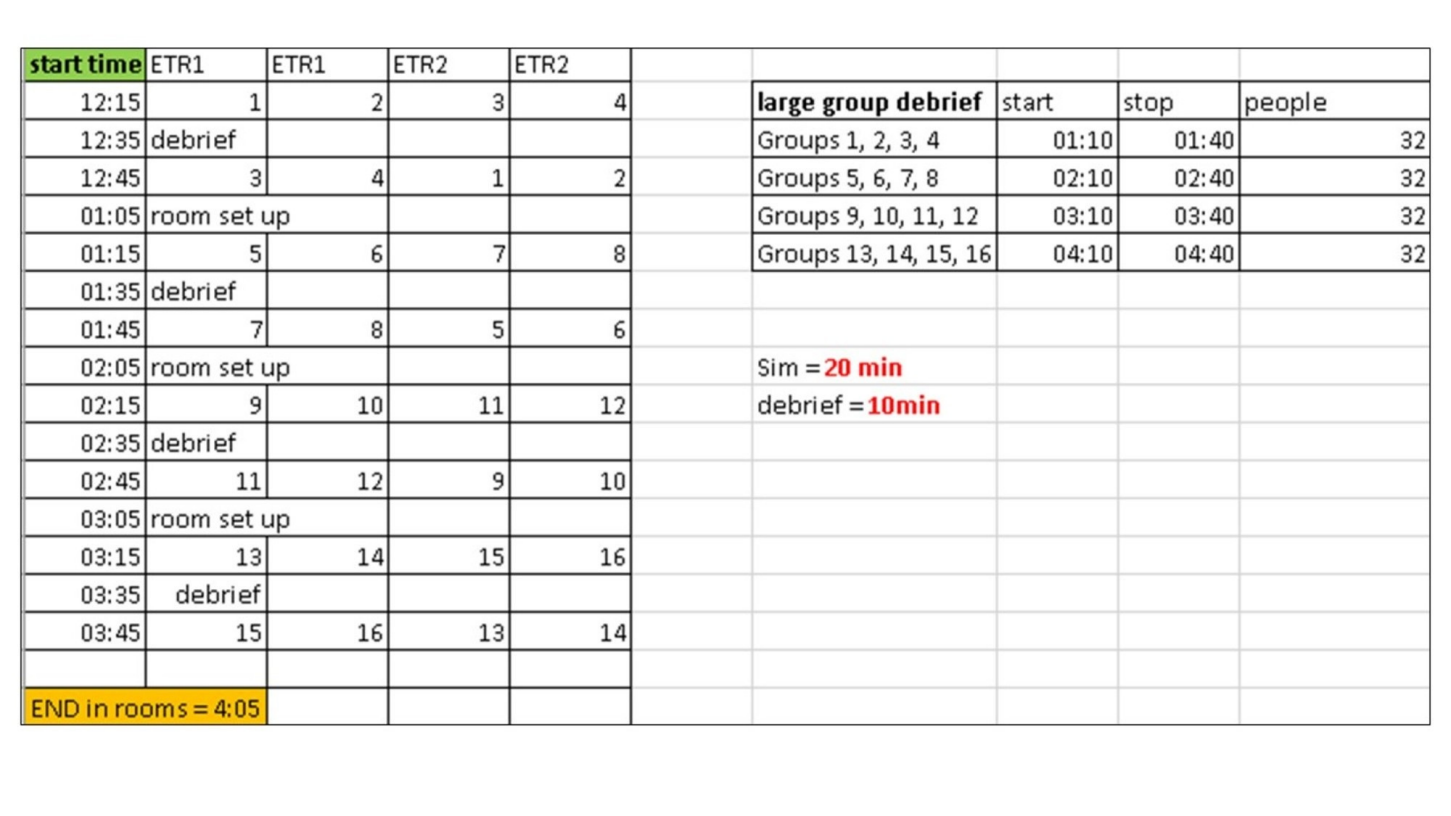
Participant flow for the escape room challenge ETR: escape the room

Simulation sessions ran throughout the afternoon. Groups were staggered, and interns were assigned a specific time to arrive at the simulation center. Prior to arrival, they received a 15-minute didactic session on the Jefferson Event Reporting System, which was led by the principal investigators (RJ). Participants received a brief primer on the concept of the escape room activity. They were then led to their respective escape rooms. The day was broken down into four 90-minute blocks (Blocks 1-4) and took place from 12 pm to 4 pm (Figure 1).

Detailed activity description

**Figure 2:**
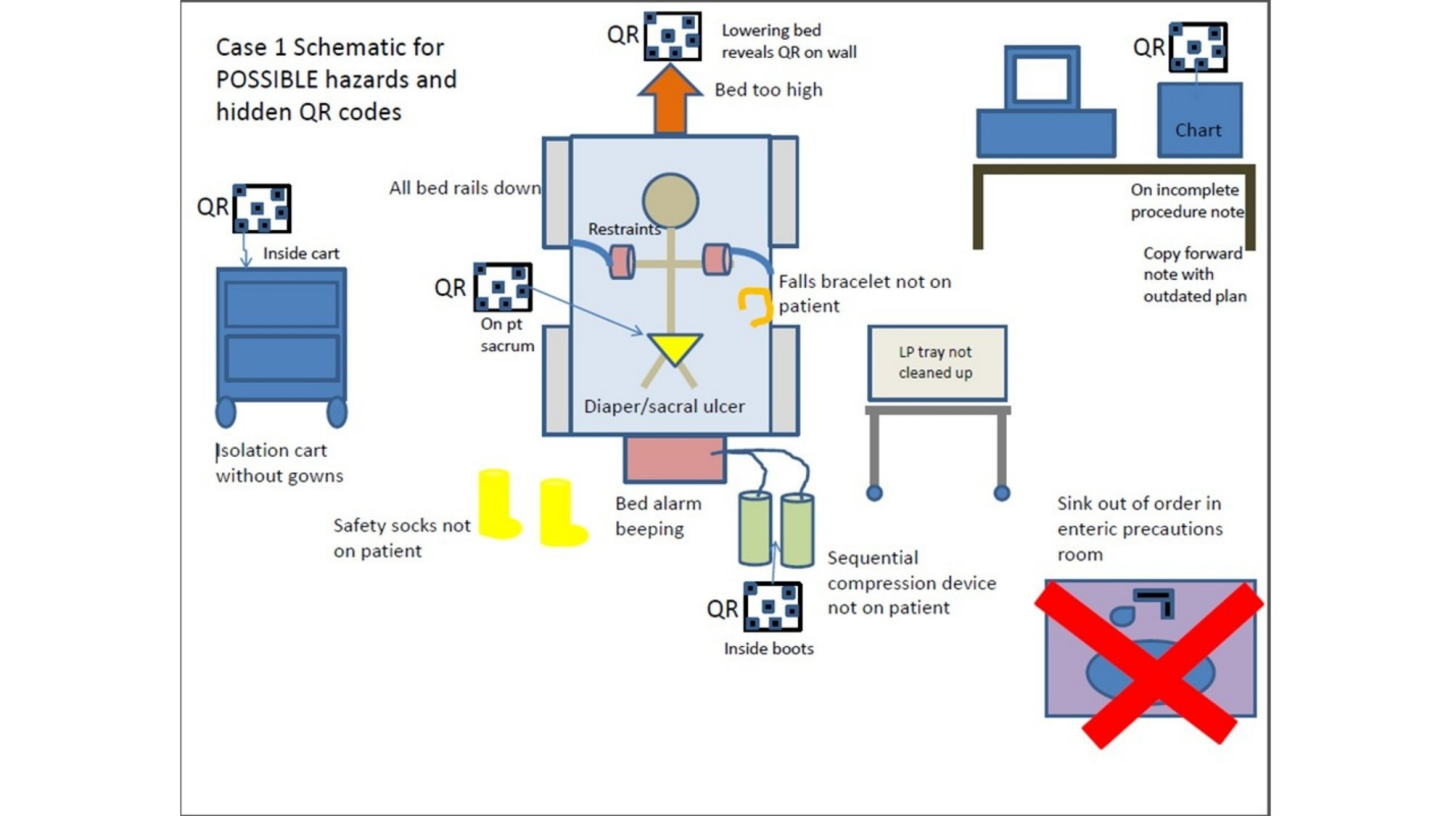
Schematic diagram of the potential clues and overlay of the simulation for Case #1 Case #1 represented a simulated inpatient hospital room with possible hazards and hidden quick response (QR) codes

**Figure 3:**
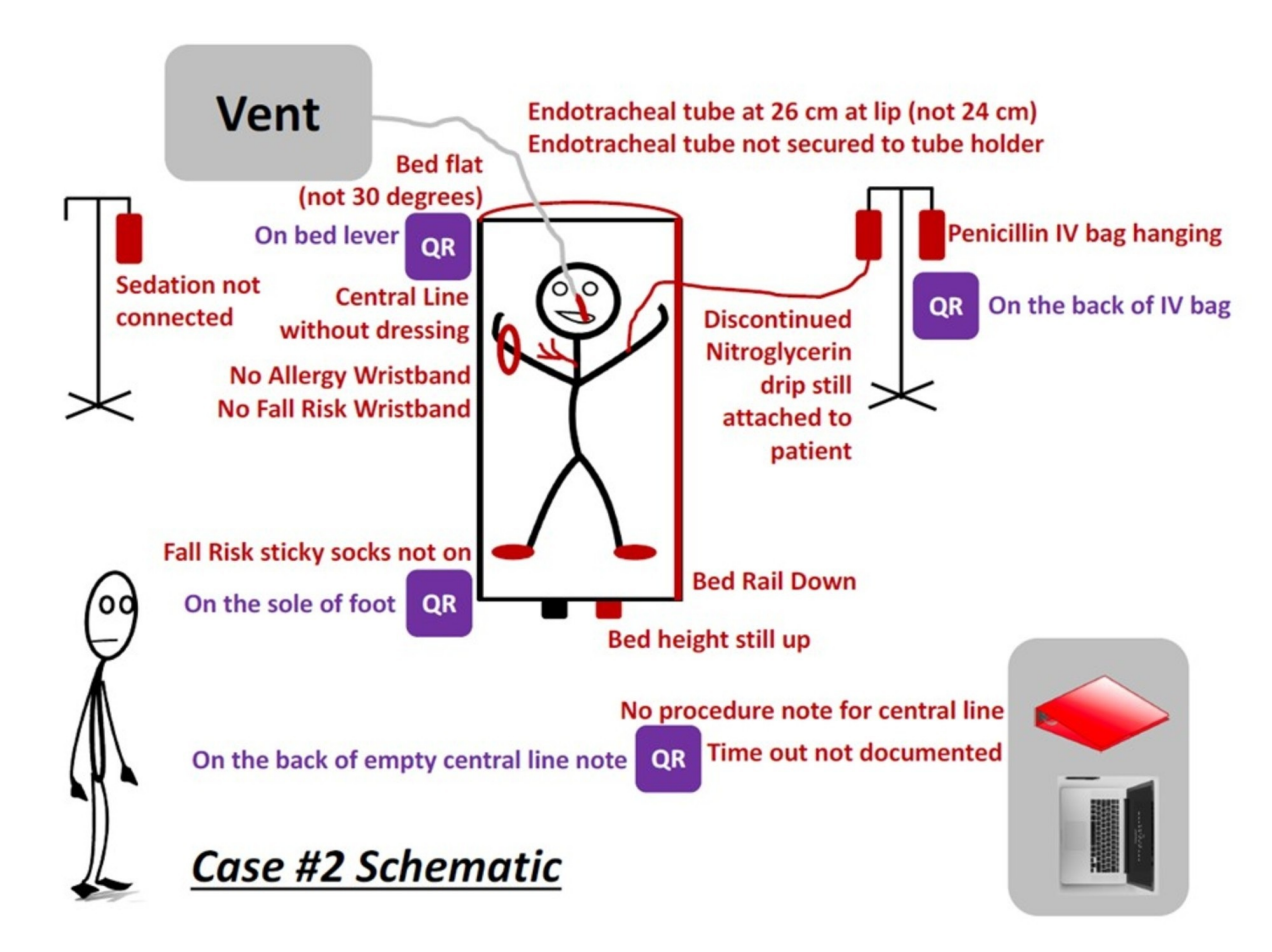
Schematic diagram of the potential clues and overlay of the simulation for case #2 Case #2 represented a simulated emergency room with possible hazards and hidden QR codes QR: quick response

Four simulation rooms were set up for the escape room activity, allowing four teams (up to 32 players) to be assessed concurrently. Half of the simulation rooms were designated as Case 1 (Internal Medicine case), and the other half were designated as Case 2 (Emergency Medicine case). Cases 1 and 2 represented two distinct simulated clinical environments with a unique set of respective challenges and patient safety hazards (Figures 2-3). Each case was developed and revised by a multi-disciplinary team composed of senior faculty clinicians of different medical specialties in order to 1) mitigate cognitive load; 2) standardize chart content; and 3) effectively link case content with the medical record, room setup, and patient safety hazards/learning goals. Each case contained a ‘patient chart’ that included: 1) an objective structured clinical encounter (OSCE) style ‘door note’ that consisted of a one-sentence introduction to frame the activity; 2) a physician note; 3) a nursing note; 4) a procedure section; 5) a clinical data section (i.e., imaging, laboratory results, electrocardiogram); and 6) an orders section. All props were obtained through the simulation center. There were no sharps or dangerous objects used. Figures 2 and 3 demonstrate different styles of illustrating the overlay of the cases with pertinent relevant patient safety hazards and props.

**Figure 4:**
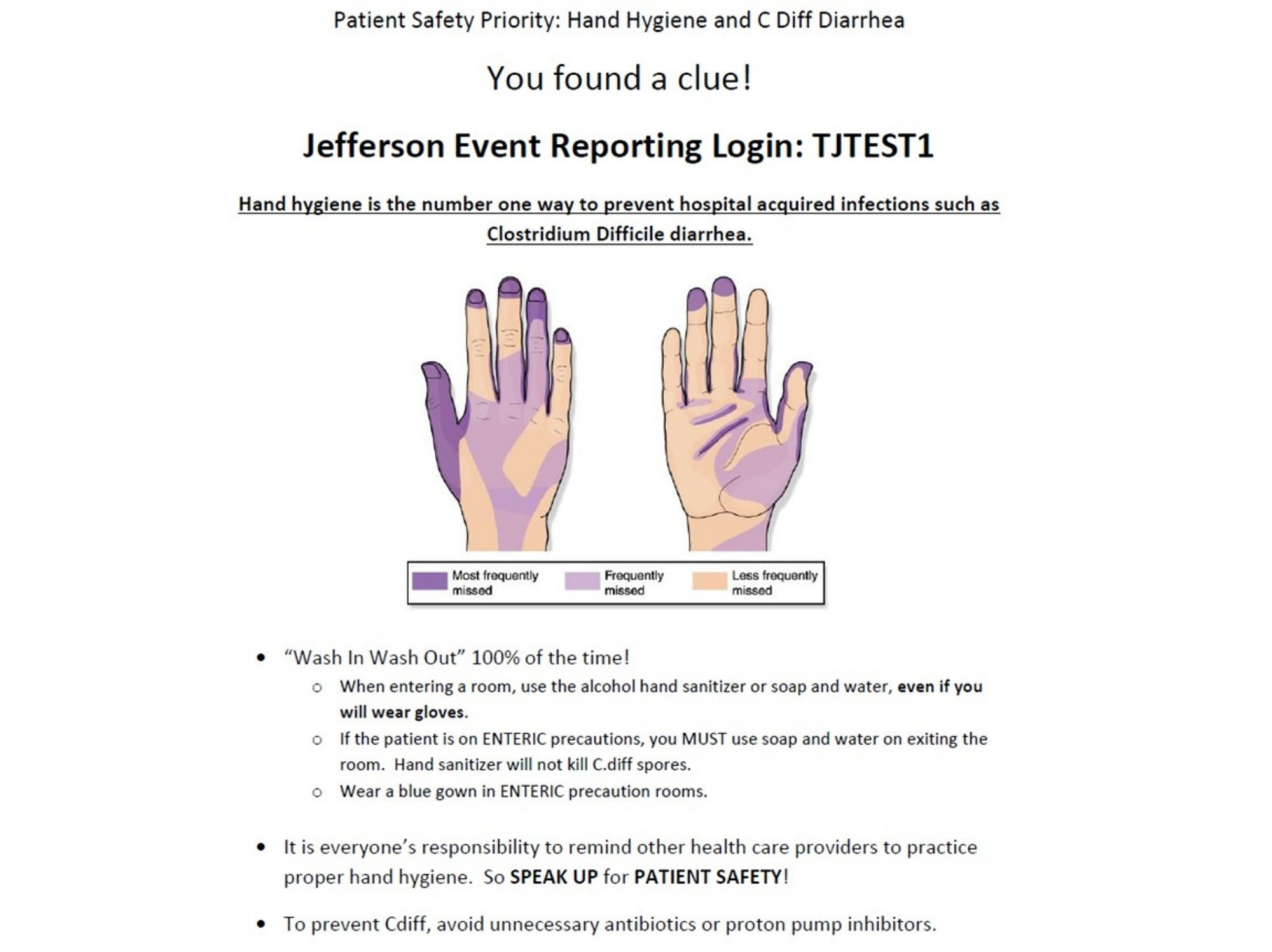
Sample QR associated clue and information Sample QR associated clue and information regarding hand hygiene as a safety hazard QR: quick response

Each team was given 20 minutes to escape the room, followed by a 10-minute team debriefing facilitated by the simulation observer. A larger 30-minute group debriefing with all of the participants during each block was also conducted after each group completed both cases. Quick Response (QR) codes were used to provide teams with instructions, clues, and prompts. QR codes can be accessed with or without paid ‘apps’ by aiming a smartphone camera directly onto the picture. Each QR code linked to a web page on a secured network with the associated information on the safety hazard or clue to be solved (Figure 4). Codes were associated with some, but not all, hazards (i.e., 3-4 hazards per room). Each code contained key information about the patient safety priority, as well as the Jefferson Event Reporting Login, password, and/or patient identifier. There were over 30 patient safety hazards shared between the two cases, such as lowered bedrails, elevated beds, wrong medication administration, missing patient-identifying wrist bands, ill-positioned breathing equipment, chart errors, soiled procedure stations, missing restraints, and ill-placed trash items. These safety hazards were selected based on a Delphi model with special consideration with implementation feasibility for this simulated study. All elements were required to be documented in the final event report. In order to successfully ‘escape the room’, participants were encouraged to practice teamwork skills (e.g., situation monitoring, closed-loop communication, leadership, and shared mental models) to solve each unique challenge.

Debriefing

**Figure 5:**
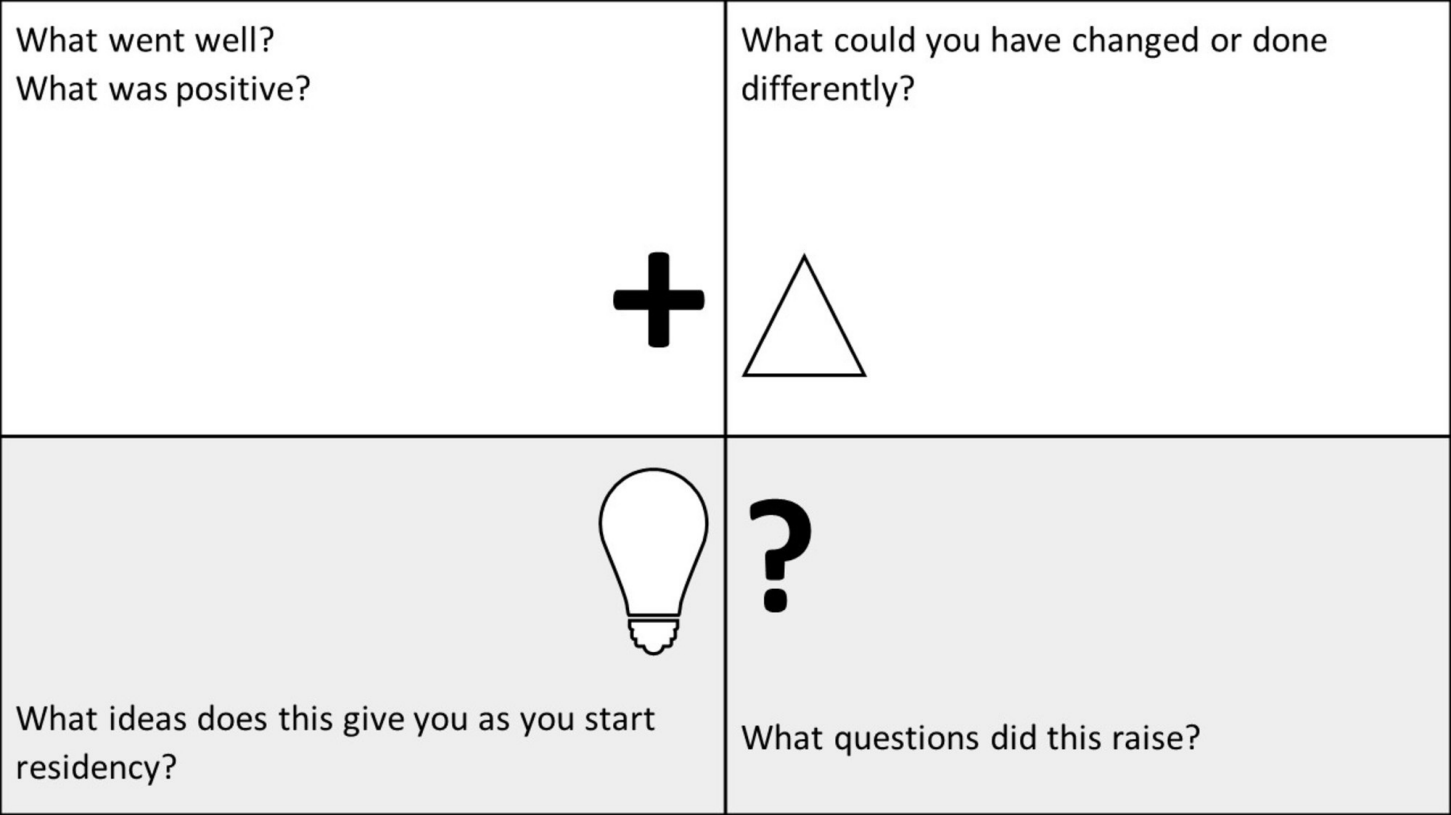
Feedback capture grid for a large group debriefing Feedback capture grid for large group debriefing. The 'plus' sign represents things that worked well. The 'triangle' shape represents what could have been done differently. The 'lightbulb' represents any new ideas. The 'question mark' represents any new questions that this activity may have raised.

After each team completed their respective escape rooms, two faculty co-facilitators debriefed participants using the feedback capture grid method (Figure 5). The feedback capture grid [16] consisted of four quadrants, each with a guiding question, such as ‘what went well?’ or ‘what would have you done differently?’ to capture different perspectives. The unique features of the feedback capture grid allow both learners and facilitators to address the strengths and potential weaknesses of the teaching activity in an organized manner, as well as provide opportunities for facilitators to address important patient safety concerns or teamwork dynamics.

Activity evaluation

Following the debriefing, participants were asked to complete a voluntary post-study survey, adapted from Brookfield’s CIQ [17]. The five-item CIQ asked participants to provide anonymous responses based on different aspects of the teaching session. This allowed investigators to model critical thinking and reflection; appreciate learning preferences; and identify potential ideas for improvement during future iterations of the activity. Responses from the questionnaire were evaluated for thematic codes through open-axial qualitative data analysis (XCZ, DP).

Results

One-hundred thirty total interns participated in the activity. All 16 teams were able to successfully escape the room within the 20-minute time limit. Thematic analysis of the post-activity CIQ survey (*n *= 102, with a 78.5% survey response rate) revealed that the learners were highly engaged by the format of the learning experience (*n *= 42) and the opportunity to identify safety threats (*n *= 30). Furthermore, participants found that role assignment (*n *= 52) and communication (*n *= 26) were the two most helpful actions needed to succeed in the activity. Participants were surprised by the overall success of the educational innovation (*n* = 45) and reported that it changed the way they perceived patient safety threats, especially identifying easily-missed errors and the large heterogeneity that exists with regards to safety threats (*n *= 7). Areas for improvement focused primarily on logistical details, where the learners identified the documentation process (*n *= 35) and the task completion (*n *= 7) of the Jefferson Event Reporting Interface as the most cumbersome, distancing and confusing elements of the activity. Table 2 illustrates a detailed thematic analysis of the CIQ questionnaire.

**Table 2:** Thematic analysis of the CIQ from the escape room event Specific CIQ questionnaires can be found in Appendix A. CIQ: critical incident questionnaire

CIQ Indices	Number of Themes Identified	Thematic Codes Generated (with frequencies, n)
Most engaged	7	Immersion in an active learning experience (42); discovering safety threats (30); debriefing (8); task performance (8); effective communication (7); teaming/team building (5); having a designated role (4)
Most distanced	6	Error reporting documentation (35); the escape room experience (16); confusion on the instructions of the activity (14); debriefing (11); not having a clear role (3); poor group communication (2)
Action most helpful	4	Role assignment (52); communication (26); feedback/debriefing (10); time-out (1)
Action most confusing	6	Task completion (7); role clarity (5); difficulty in identifying patient safety threats (2); challenges in communication (2); event reporting (1); team dynamics (1)
Surprised you most	4	Success of the educational innovation (45); paradigm shift in how patient safety threats are viewed (18); value of teamwork (12); error reporting (3)

## Discussion

The authors propose that escape rooms can be utilized to teach patient safety hazards and serve as an effective teambuilding activity. Open-axial qualitative analysis of the participants CIQ responses revealed that residents recognized the importance of identifying patient safety hazards and felt fully immersed in this innovative method for teaching patient safety and team-building. Additional aspects of the program that residents appreciated included the importance of closed-loop communication, team-work building skills (i.e. taking notes, assigning a team leader), and formal debriefing. Participants also identified areas of improvement that distanced themselves from completing the objectives, such as complex event reporting, confusing instructions (e.g. how to escape the room), and unclear role clarity among teams, which was limited to the team dynamic of the individual groups, rather than the overall escape room activity. Participants, however, were appreciative that efforts were made to incorporate the institution’s official patient safety reporting system into the simulation experience, as it allowed them to feel more confident and comfortable with navigating the software for future use. In order to address these issues, future iteration includes increased instruction time during the 15-minute didactic session on the goals of the escape room activities, as well as providing the teams extra preparation time to discuss teamwork tactics or leadership selection before entering the rooms. 

There are several limitations worth noting. The simulations only included some of the common patient errors found in the inpatient and ED setting. The inclusion of these patient safety issues can unintentionally limit learners’ focus on these case-specific details. Furthermore, while we encouraged event reporting through active practice within the simulations, additional studies will need to be performed on this cohort to see whether this intervention will lead to an increased number of reporting rates over time.

## Conclusions

The escape room patient-safety activity was well received by incoming resident physicians at the Thomas Jefferson University. The activity allowed learners to actively engage in a fun, non-threatening, low-stakes activity that illustrated the expansive world of patient safety hazards, as well as providing them with the opportunity to document event reports in real time. Next steps will include tracking the quantity of error reports entered by this cohort longitudinally to determine the effectiveness of this educational intervention on behavioral change.

## Appendices

Appendix A – Critical Incident Questionnaire [16].

1. At what moment during the activity did you feel most engaged with what was happening?

2. At what moment during the activity did you feel most distanced from what was happening?

3. What action did anyone (i.e., faculty member or peer) take during the activity that you found most affirming and helpful?

4. What action did anyone (i.e., faculty or peer) take during the activity that you found most puzzling or confusing?

5. What about the activity this week surprised you the most? (This could be something about your own reactions to what went on, or something that someone did, or anything else that occurs to you.)
